# *Staphylococcus aureus In Vitro* Secretion of Alpha Toxin (hla) Correlates with the Affiliation to Clonal Complexes

**DOI:** 10.1371/journal.pone.0100427

**Published:** 2014-06-18

**Authors:** Stefan Monecke, Elke Müller, Joseph Büchler, Bettina Stieber, Ralf Ehricht

**Affiliations:** 1 Alere Technologies GmbH, Jena, Germany; 2 Institute for Medical Microbiology and Hygiene, Technische Universität Dresden, Dresden, Germany; 3 Alere San Diego, Inc., San Diego, California, United States of America; Columbia University, United States of America

## Abstract

The alpha toxin of *Staphylococcus aureus* is a pore forming toxin that penetrates host cell membranes causing osmotic swelling, rupture, lysis and subsequently cell death. Haemolysin alpha is toxic to a wide range of different mammalian cells; i.e., neurotoxic, dermonecrotic, haemolytic, and it can cause lethality in a wide variety of animals. In this study, the *in vitro* alpha toxin production of 648 previously genotyped isolates of *S. aureus* was measured quantitatively using antibody microarrays. Isolates originated from medical and veterinary settings and were selected in order to represent diverse clonal complexes and defined clinical conditions. Generally, the production of alpha toxin *in vitro* is related to the clonal complex affiliation. For clonal complexes CC22, CC30, CC45, CC479, CC705 and others, invariably no alpha toxin production was noted under the given *in vitro* conditions, while others, such as CC1, CC5, CC8, CC15 or CC96 secreted variable or high levels of alpha toxin. There was no correlation between alpha toxin yield and clinical course of the disease, or between alpha toxin yield and host species.

## Introduction

*Staphylococcus (S.) aureus* is a gram-positive coccus that is an important commensal bacterium and pathogen in both, animals and humans. Some 30% of a healthy human population carries *S. aureus* asymptomatically in the anterior mucosa of their noses. Animals of several species also might be colonized or infected. *S. aureus* also can cause a variety of different infections including localised skin and soft tissue infections, more severe conditions such as osteomyelitis or pneumonia and life-threatening endocarditis or septicaemia. This bacterium can also trigger toxin-mediated diseases such as food intoxication, toxic shock and scalded skin syndromes. It is known to harbour, beside genes associated with drug resistance and adhesion to host tissues etc., a complex array of virulence factors that includes superantigens (toxic shock syndrome toxin, *tst1*, and some 30 enterotoxin genes), exfoliative toxins, proteins that interfere with various functions of the host immune system (complement and chemotaxis inhibitors *etc.*), leukocidins (*i.e.*, toxins that specifically destroy white blood cells by formation of polymeric pores in cell membranes) and different haemolysins. The latter are proteins that lyse amongst others red blood cells, allowing for instance the bacterium to scavenge iron compounds. In *S. aureus*, there are three major, well characterised haemolysins, named alpha, beta, and gamma, as well as additional genes that are assumed to encode for additional haemolysins (such as BA000017.4: locus tag SAV0919; base positions 962,930 to 963,970 or CP000046.1: locus tag SACOL2160; base positions 2,239,231 to 2,239,914).

The alpha toxin (also known as haemolysin alpha) is encoded by the *hla* gene (BA000018.3: locus tag SA1007; base positions 1,140,562 to 1,141,521). To our best knowledge, this gene can be found in all *S. aureus* strains and isolates, and it is always situated at approximately the same localisation within the staphylococcal chromosome, *i.e*., around base positions 1,110,000 to 1,230,000 in a genome of about 2,743,000 to 3,043,000 base pairs of total length. There is a relatively small sequence variation of *hla* alleles (see File S1) across the different clonal complexes (CC; as defined by Multilocus Sequence Typing, MLST, [1]) of *S. aureus*. Compared to the DNA sequence of the CC5 strain N315 (BA000018.3) as reference there are, for instance, five nucleotide exchanges (out of 960 positions) in the CC8 strain COL (CP000046), 50 nucleotide exchanges in the CC30 strain Sanger MRSA 252 (BX571856) and 137 for the CC75 strain MSHR1132 (FR821777), a strain that is so different from other *S. aureus* that it has been proposed to regard it as species on its own [2]. These nucleotide exchanges result for the three strains in two, three and 36 amino acid exchanges compared to N315, respectively.

The alpha toxin is a pore forming toxin. The pore is a polymeric ring with a diameter of 1–2 nm comprising of seven 33 kDa protein molecules that penetrates in host cell membranes causing, osmotic swelling, rupture, lysis and subsequently cell death. Haemolysin alpha is toxic to a wide range of different mammalian cells; *i.e.*, neurotoxic, dermonecrotic, haemolytic, and it can cause lethality in a wide variety of animals. Its toxic effects include activation of the arachidinic metabolism in endothelial cells due to Ca^2+^ influx, activation of cellular nucleases [3] and resulting apoptosis [4], activation of the autophagic pathway in case of intracellular presence of *S. aureus* [5], release of procoagulatory factors due to Ca^2+^ influx in platelets, vasoconstriction associated with a liberation of thromboxane A(2) and prostacyclin [6], and an increase of vascular permeability that might lead to pulmonary oedema and adult respiratory distress syndrome. Experimental studies indicated alpha toxin to be an important virulence factor in both rabbit and murine models of keratitis [7] as well as in pneumonia [8], [9] and superinfection of influenza [10]. Alpha toxin is regulated by both, the *agr* and *saeR/S* systems as the deletion of *saeRS*, and, to a lesser extent, *agr* resulted in its attenuated expression [11]. Similar observations were also described with regard to *sarA/Z* [12] indicating that alpha toxin, as well as other exotoxins such as Panton-Valentine leukocidin and proteases are up-regulated during the dissemination phase of *S. aureus* infections but down-regulated during a stationary phase when factors prevail that are associated with biofilm formation and adhesion.

The *hla* gene is present essentially in all isolates and lineages of *S. aureus.* Invasive or non-invasive isolates thus do not differ in the mere presence or absence of the gene, and this warrants the study of the expression or regulation of the toxin. Therefore, the aim of the study was to develop, establish and use a simple and robust antibody-based system for the quantitative measurement of alpha toxin in *S. aureus* cultures.

## Materials and Methods

### Strains

In this study, 648 isolates and reference strains were tested. They originated from medical and veterinary settings and were selected in order to represent diverse clonal complexes and defined clinical conditions. All isolates, were previously genotyped using the StaphyType Kit (Alere Technologies, Jena, Germany) as described in detail before [13], [14]. Using this method, relevant genes as typing-, virulence- and resistance marker were determined and isolates were assigned to CCs and strains. Characterisation and hybridisation profiles of all lineages and most isolates have been described previously [13], [14], [15], [16], [17], [18], [19], [20], [21], [22], [23], [24], [25].

### Culture Conditions

Strains were cultured on Columbia blood agar (agar basis Oxoid, CM331 and sheep blood OXOID, FSR1055) and incubated for 24 hrs at 37°C. One loop of bacterial material was inoculated into 65 µl 100 mM NaOH, vortexed and incubated for 5 min at room temperature (RT). This procedure yielded slightly better results than a suspension in PBS (data not shown), presumably due to lysis of cells and release of intracellularly stored alpha toxin. Then, 65 µl of phosphate solution (pH 5,5; 1M di-sodium hydrogen phosphate, 1M sodium-di-hydrogen phosphate) was added for neutralisation and vortexed. The mixture was diluted 1∶10 in buffer [1x PBS; 0.05% Tween; 1% FCS; 0.25% Triton X-100] for further analysis.

### Antibodies

Alpha Toxin (HT101; Toxin Technology, Sarasota, Florida, USA) from the *S. aureus* strain Wood 46 (CC97-MSSA) was used to generate monoclonal antibodies via phage display. Following immunisation of mice, mRNA from their B-cells was isolated and amplified. Resulting cDNA, specific for the antigen-binding parts of antibodies, was ligated into bacteriophages and transformed into *E. coli*. Resulting antibodies were purified and characterized for specificity and sensitivity by ELISA microtiterstrip-mounted protein microarrays. For further experiments, three different alpha toxin antibodies were immobilised on the array at nine different concentrations.

### Array Procedures and Detection

First, the protein microarrays were pre-washed and blocked. Arrays were incubated with 150 µl buffer (as above) at 37°C for 5 min at 400 rpm on a shaker, followed by 100 µl blocking solution (10% foetal calf serum) for 5 min at 37°C and 300 rpm. Then, 100 µl of the suspended bacteria was added and incubated at 37°C for 30 min at 300 rpm.

A secondary antibody was labelled with biotin (Sulf-NHS-LC-Biotin; Pierce, Bonn, Germany) and used (*ca.* 0.2 ng/µl, 100 µl, 37°C for 30 min at 300 rpm) to detect bound proteins followed by addition of streptavidin-horseradish peroxidase (HRP; 0,5 ng/µl, 100 µl, 15 min at 37°C and 300 rpm). The latter induced the local precipitation of the added dye, tetramethylbenzidine (TMB; 10 min without shaking at room temperature). The precipitation reaction was stopped by removing the substrate [26], [27], [28].

### Analysis

Array images were taken by the ArrayMate reading device (Alere Technologies, Jena, Germany) and analysed using IconoClust software according to manufacturer’s instructions. The quantitative analysis based on a set of previously established calibration- and reference-experiments with three alpha toxin antibodies each spotted at nine different concentrations. For calibration curves, alpha toxin (1 mg/ml; Toxin Technology) was tested at different defined concentrations (0.1 ng/ml, 0.2 ng/ml, 0.4 ng/ml, 0.6 ng/ml, 0.8 ng/ml, 1.0 ng/ml, 2.0 ng/ml, 3.0 ng/ml, 4.0 ng/ml, 6.0 ng/ml, 8.0 ng/ml, 10.0 ng/ml). Resulting signal intensities of each single spot were correlated to the known antigen concentrations. Thus, standard curves were generated for each concentration of each antibody. In the following experiments, unknown concentrations of alpha toxin were determined by mapping the intensity of array spots on the previously established calibration curves. This approach has previously been described in detail for another staphylococcal toxin, PVL [29].

## Results

### Yield of Alpha Toxin and Affiliation to Clonal Complexes

Generally, the yield of alpha toxin appears to be related to the CC affiliation. For a detailed overview, see Table 1. For clonal complexes CC22, CC30, CC45, CC479, CC705 and others consistently no alpha toxin production, or very low levels thereof, was noted under the given *in vitro* conditions. Isolates from other lineages (such as CC1, CC5, CC8, CC15 or CC96) secreted variable to high levels of alpha toxin. Only few isolates (one or two from CC398, CC59, respectively) yielded positive results despite affiliation to a CC that normally did not produce detectable amounts of alpha toxin; or lacked alpha toxin production while belonging to an otherwise positive ST (one isolate from ST72).

**Table 1:** Alpha Toxin yields by CC/ST affiliation.

Clonal Complex/Sequence Type	Testedisolates	Hostspecies	Mean(ng/ml)	Median(ng/ml)	Range(ng/ml)			Standard deviation(ng/ml)
**CC1**	40	Human	8.2	6.5	0.5	…	31.7	6.7
**CC1 (ST567)**	1	Human	6.2	6.2	6.3	…	6.2	–
**CC1 (ST573/772)**	8	Human	0.0	0.0	0.0	…	0.0	0.0
**CC5**	46	Human	5.3	1.1	0.0	…	32.1	7.9
**CC5** **(** ***excluding*** ** ST228-MRSA-I)**	33	Human	7.6	5.7	0.0	…	32.1	8.5
**CC5** **(ST228-MRSA-I)**	14	Human	0.0	0.0	0.0	…	0.0	0.0
**CC6**	5	Human (4),camel (1)	12.0	11.3	0.5	…	27.6	11.5
**CC7**	10	Human	21.1	13.5	4.5	…	78.4	22.2
**CC7 (ST1048)**	1	Human	8.9	8.9	8.9	…	8.9	–
**CC8**	70	Human	12.7	11.4	0.0	…	41.3	9.7
**CC8 (ST72)**	10	Human	8.8	7.9	0.0	…	16.6	5.6
**CC8 (ST239)**	9	Human	3.3	2.8	0.5	…	6.6	2.2
**CC9**	8	Human	0.0	0.0	0.0	…	0.0	0.0
**CC9 (ST834)**	5	Human	9.5	12.3	0.5	…	13.1	5.4
**CC10**	6	Human	2.1	0.2	0.0	…	6.4	3.1
**CC12**	11	Human	8.0	4.8	1.1	…	30.8	8.9
**CC15**	26	Human	17.0	7.2	0.0	…	72.8	22.4
**CC20**	8	Human (5),cattle (3)	3.0	0.5	0.0	…	12.4	4.6
**CC22**	34	Human (33),cattle (1)	0.0	0.0	0.0	…	0.0	0.0
**CC25**	14	Human	6.8	6.0	0.0	…	18.6	6.5
**CC30**	47	Human	0.2	0.0	0.0	…	2.4	0.5
**CC30 (ST34/42)**	8	Human	0.0	0.0	0.0	…	0.0	0.0
**CC45 (** ***agr*** ** I)**	40	Human	0.0	0.0	0.0	…	0.0	0.0
**CC45 (** ***agr*** ** IV)**	2	Human	0.0	0.0	0.0	…	0.0	0.0
**CC49**	3	Human	0.0	0.0	0.0	…	0.0	0.0
**CC50**	5	Human	0.0	0.0	0.0	…	0.0	0.0
**CC59**	15	Human	1.9	0.0	0.0	…	15.9	4.5
**CC80**	6	Human	10.6	6.2	0.5	…	28.4	11.7
**CC88**	3	Human (2),cattle (1)	0.0	0.0	0.0	…	0.0	0.0
**ST93**	4	Human	0.0	0.0	0.0	…	0.0	0.0
**CC96**	5	Human	22.9	17.7	0.5	…	64.0	24.8
**CC97**	12	Human (11),Moose (1)	12.1	11.5	2.0	…	30.6	7.6
**CC97 (ST71)**	4	Cattle	6.9	7.1	4.8	…	8.7	1.6
**CC101**	6	Human	0.0	0.0	0.0	…	0.0	0.0
**CC121**	16	Human	0.30	0.0	0.0	…	1.8	0.5
**CC130**	2	Human, Hedgehog	0.0	0.0	0.0	…	0.0	0.0
**CC133**	4	Human (2),Goat (1), Cattle (1)	0.0	0.0	0.0	…	0.0	0.0
**ST140**	3	Human	0.3	0.5	0.0	…	0.5	0.5
**CC152**	7	Human	0.0	0.0	0.0	…	0.0	0.0
**CC182**	4	Human	0.0	0.0	0.0	…	0.0	0.0
**CC188**	12	Human (6),cattle/water buffalo (6)	10.5	8.6	0.0	…	30.6	10.5
**CC188 (ST1774)**	4	Human	8.4	9.3	0.5	…	15.6	7.1
**ST350**	4	Human (1),Cattle (2), Dog (1)	1.5	0.5	0.0	…	4.9	2.3
**CC361**	5	Human (3), Cattle (2)	0.0	0.0	0.0	…	0.0	0.0
**CC395**	9	Human	1.7	1.2	0.0	…	5.9	1.8
**CC398**	19	Human (8), Pig (1),Turkey (2), Cattle (6)	0.32	0.0	0.0	…	6.2	1.4
**CC398 (ST291/813)**	17	Human (4), cattle/waterbuffalo (13)	4.3	1.2	0.0	…	23.5	6.5
**ST425**	6	Human (2), badger (2),cattle (1), Red deer (1),	0.0	0.0	0.0	…	0.0	0.0
**CC479**	4	Cattle	0.0	0.0	0.0	…	0.0	0.0
**CC509**	3	Human	0.0	0.0	0.0	…	0.0	0.0
**CC522**	4	Goat (2), sheep(2)	0.0	0.0	0.0	…	0.0	0.0
**CC599**	1	Human	0.0	0.0	0.0	…	0.0	–
**CC692**	4	Common magpie,Green woodpecker, Tawny owl,White-tailed eagle	10.7	5.6	3.8	…	27.8	11.4
**CC705**	16	Cattle	0.0	0.0	0.0	…	0.0	0.0
**CC707**	5	Human (4),reindeer (1)	0.0	0.0	0.0	…	0.0	0.0
**CC779**	3	Human	0.0	0.0	0.0	…	0.0	0.0
**ST816**	1	Dog	3.4	3.4	3.4	…	3.4	–
**CC913**	1	Human	28.3	28.3	28.3	…	28.3	–
**CC1021**	3	Human	0.3	0.5	0.0	…	0.5	0.3
**ST1093**	3	Human	1.7	1.7	1.3	…	2.1	0.4
**ST1290/2481**	1	Human	1.3	1.30	1.3	…	1.3	–
**CC1943**	1	Human	0.0	0.0	0.0	…	0.0	–
**ST2279**	2	Lynx, Reindeer(1 each)	0.0	0.0	0.0	…	0.0	0.0
**ST2425**	2	Brown hare	2.7	2.7	0.5	…	4.9	3.1
**ST2691**	2	Moose	0.0	0.0	0.0	…	0.0	0.0
**“** ***S. argenteus*** **” (ST75, ST883)**	7	Human	0.0	0.0	0.0	…	0.0	0.0

Certain sequence types present with very different DNA array hybridisation patterns (different *agr* group, different capsule type, and other alleles of *ssl* or MSCRAMM genes) than the CC they are assigned to according to sequence based algorithms (BURST; see http://saureus.mlst.net/eburst/). Some of them are known to originate from large scale chromosomal replacements [30]. In some instances, such deviant STs show essentially the same alpha toxin results as the CC they are derived from; examples being CC1 (ST567), CC7 (ST1048), CC8 (ST72), CC30 (ST34/42), CC97 (ST71) or CC188 (ST1774). In a few instances, there were differences to the parental lineage. This included CC398 (ST291/813) strains where the alpha toxin yield was higher than in other CC398. For the mosaic lineage CC8 (ST239) that comprises genome segments of both, CC8 and CC30 origin [30], less toxin was measured than for CC8 strains but more than for CC30. CC1 (ST573/772) lacked *hla* expression while other CC1 isolates were positive. CC9 (ST834) was a strong alpha toxin producer, while other CC9 isolates invariably were negative.

There was one clearly defined strain that differed in alpha toxin *in vitro* production from other strains belonging to the same clonal complex. 14 isolates of ST228-MRSA-I, “South German Epidemic MRSA”, coming from different geographic regions (South-Eastern Germany, Malta, Switzerland and Denmark) were all alpha toxin negative while the parental lineage, CC5, usually is clearly positive.

### Alpha Toxin and Agr Group Affiliation

To test the null-hypothesis that the HLA concentrations are the same in each *agr-*group we performed the Kruskal-Wallis rank sum test. Here, in contrast to the analysis of variance (ANOVA), it is not required that the data within the groups are normally distributed and have the same standard deviation. As for the differences between *agr* groups, the Kruskal-Wallis test shows a probability below 0.01 for a true null-hypothesis (Chi-Square = 15.5, Degrees of freedom = 4, Probability of a true null-hypothesis = 0.003777). Therefore, it is likely that the HLA concentrations are truly different between the different *agr* groups. Between *agr* groups I, II and III, there is no major difference in mean/median alpha toxin yields (Table 2); and all these groups harbour clonal complexes that are strong as well as poor producers. The yield of *agr* group IV isolates was clearly lower, there was no strongly producing lineage within this group and isolate numbers also were low compared to the others. The isolates which were not assignable to *agr* groups all belonged to the “*S. argenteus*” lineage (ST75/ST883), and failed to produce detectable alpha toxin.

**Table 2:** Alpha Toxin yields and *agr* group affiliation.

	Testedisolates	Mean(ng/ml)	Median(ng/ml)	Range(ng/ml)			Standard deviation(ng/ml)
***agr*** ** Group I**	341	5.5	0.5	0.0	…	78.4	8.9
***agr*** ** Group II**	142	5.8	0.5	0.0	…	72.8	12.4
***agr*** ** Group III**	131	4.0	0.5	0.0	…	64.0	8.2
***agr*** ** Group IV**	27	0.4	0.0	0.0	…	4.9	1.0
**“** ***S. argenteus*** **” (ST75, ST883)**	7	0.0	0.0	0.0	…	0.0	0.0

### Alpha Toxin and Host Species

No correlation of alpha toxin production and host species was noted. Bovine isolates ranged from negative to strongly positive, depending not on bovine origin but on CC affiliation with isolates belonging, *e.g*., to CC398, CC479, CC705 being negative while CC398 (ST291/813) and CC97 (ST71) being positive. For other host species, isolate numbers were low, but apparently the general picture is similar. For instance, two moose (*Alces alces*) isolates belonging to ST2691 were negative while another two assigned to CC97 were positive. CC398 from turkey yielded negative tests, while CC692 from other birds (magpie, tawny owl, green woodpecker, white-tailed eagle; *Pica pica, Strix aluco, Picus viridis, Haliaeetus albicilla*) were positive.

### Alpha Toxin, Outcome of Disease and Clinical Syndromes

Animal isolates and isolates without known diagnosis were excluded from this analysis. No correlation between *in vitro* alpha toxin yield and fatal outcome was observed (Table 3).

**Table 3:** Alpha Toxin yields and clinical outcome (veterinary isolates excluded).

	Testedisolates	Mean(ng/ml)	Median(ng/ml)	Range(ng/ml)			Standarddeviation (ng/ml)
**All cases**	428	5.55	0.5	0.0	…	72.8	10.1
**Fatal cases**	34	3.01	0.2	0.0	…	19.2	4.8
**Surviving; or outcome not recorded**	394	5.77	0.5	0.0	…	72.8	10.4

## Discussion

The assay described herein allows to rapidly and quantitatively measuring alpha toxin from staphylococcal cultures. This allows studying expression with regard to strain- or CC affiliations, but also with regard to regulation under different growth conditions in the presence of antibiotics, *etc.* It could also be expanded by spotting additional antibodies for other exotoxins such as PVL [29] on the same array, facilitating simultaneous measurements of several virulence factors.

The most surprising aspect in this work was that the *in vitro* production of alpha haemolysin normally strongly correlates with affiliation to clonal complexes. This can theoretically be explained at least by two different assumptions. One is a possible presence of allelic variants resulting in proteins that might less efficiently be recognised by the antibodies used. The second one could be that expression and/or regulation indeed vary, depending on the affiliation to phylogenetic lineages.

The first possible explanation could be true in CC75/“*S. argenteus*”. As mentioned above, a genome sequence from this lineage shows several differences compared to other *S. aureus* sequences. Thus antibodies specific for other alpha toxin variants might false-negative results or give a false impression of low toxin levels. Raising monoclonal antibodies specifically for CC75/“*S. argenteus*” alpha toxin might resolve this issue in future. For other lineages, allelic differences between *hla* sequences of different clonal complexes are rather small (see Introduction and File S1), even less when regarding protein rather than DNA sequences. Allelic variations are so not a likely cause for the different alpha toxin measurements for these lineages. It was observed that CC395 was usually alpha toxin positive while CC22, CC30, CC45 and CC398 are negative in the described test. However, a published CC395 sequence (AGRO01000049∶167700 to 168659) is identical to sequences from several CC45 strains and virtually identical to CC22, CC30 and CC398 sequences. The variations within major complexes for which many sequences are available appear to be larger than between complexes (for an example, CC8, see File S1). Besides, the impact of allelic variation is minimised by the use of three monoclonal antibodies.

The second possible explanation is a lineage specific expression. Such a relation of *hla* expression to clonal complex background could also be assumed analysing data from an earlier study [31] that measured toxin expressions in pandemic MRSA strains using an entirely different RNA-based approach, *i.e*, quantitative reverse-transcription PCR. These authors observed a lack of *hla* expression in CC30 strains USA200 and USA1100 (CC30/ST36-MRSA-II and PVL-positive CC30-MRSA-IV). They observed intermediate levels of *hla* expression for “USA400” (CC1), “USA100” (CC5), “USA1100” (CC59) and in a ST72 strain as well as high levels in “USA300” and “USA500” (CC8) as well as in a ST80 strain. This is in quite a good accordance to our observation despite the use of an entirely different method as well as of another growth medium.

The differences in alpha toxin expression are not related to *agr* types since strong as well as poor producers are can both be found within one *agr* group. An exemption might be *agr* group IV, which, however, is genetically much less diverse than *agr* groups I-III comprising only two major complexes (CC50 and CC121). In normally poorly producing lineages (such as CC59), occasionally strong producers can be observed. This could indicate that the variability of alpha toxin levels within one lineage was wider than the relatively few experiments might suggest. It could also imply that a short-term adaption to external selective pressures (immunity in a specific host, exposure to drugs) could modify the modus of regulation and expression.

The absence of detectable alpha toxin production in some CC30 strains (*e.g*., ST36-MRSA-II, MRSA252, GenBank BX571856) could possibly be attributed to a mutation resulting in a TAG stop codon within the *hla* sequence [32], see File S1 (position nr. 338). There might also be similar mutations in strains for which no sequences are yet known. However, even in CC30 there are strains that do not show this mutation [33], [34], see File S1, so that other reasons for absence or low levels of alpha toxin production must also play a role.

Most interestingly, isolates of one epidemic strain (CC5/ST228-MRSA-I, “South German Epidemic Strain”) consistently proved to alpha toxin negative although that strain belongs to a strongly producing lineage (CC5). Given that this strain is currently superseded by others [35], one might speculate if this deficiency might play a role in its demise. The reason for an *in vitro* absence of alpha toxin is not yet known. It is likely not related to sequence variations of *hla*, *i.e*., a possible presence of an epitope altered beyond recognition, as several *hla* sequences (GenBank accession numbers HE579059, HE579061, HE579063, HE579065, HE579067, HE579069, HE579073) of ST228-MRSA-I are identical to N315 and other CC5 sequences. There is one single nucleotide polymorphism in the published *agrC*-II sequences of this strain compared to N315 (A instead of T in position 91), but if this was indeed related to a lack of alpha toxin production warrants further study.

It was also observed that alpha toxin production apparently did not correlate with host species, with alpha toxin production being detected also in isolates from non-human hosts. Although further, more systematic studies on alpha toxin in animal isolates of *S, aureus* are needed, this could indicate a crucial role of this toxin for *S. aureus* as opportunistic pathogen in a wide range of hosts. This observation could also be in accordance to the apparent promiscuity of alpha toxin with regard to target cells.

Finally, the clinical course of staphylococcal disease and the *in vitro* production of alpha toxin did not correlate with isolates from fatal cases and survivors secreting essentially identical amounts of the toxin. This could indicate that i) the *in vitro* and *in vivo* situations cannot be compared, or ii) even small concentrations of alpha toxin are sufficient to fulfil its pathophysiological role so that high level producers just produce in excess, or iii) that the effect of alpha toxin is complemented or superseded by other haemolysins and exotoxins and that it is only one factor among many others. However, its presence in *S. aureus* appears to be crucial for the virulence of that species.

## Supporting Information

File S1 Alignment of *hla* sequences.
(FAS)
